# Polish adaptation of multisource assessment of children’s social competence

**DOI:** 10.1038/s41598-023-39292-2

**Published:** 2023-07-26

**Authors:** Agata Wiza, Agnieszka Koszałka-Silska, Maciej Jaguszewski, Magdalena Lewandowska

**Affiliations:** 1 Department of Pedagogy, Poznan University of Physical Education, Poznan, Poland; 2 Calculation Centre, Poznan University of Physical Education, Poznan, Poland

**Keywords:** Psychology, Health care

## Abstract

This study aimed to adapt the Multisource Assessment of Social Competence Scale for the Polish population. The population examined in the study included only Polish participants of European (Caucasian) ancestry. The tool is composed of two scales, Prosocial and Antisocial, and four subscales. For the purpose of adaptation, children’s social competence was evaluated based on their own and their parents’ perspective. The sample consisted of children aged 9–15 (n = 253) and their parents (n = 248), with boys and girls accounting for 43% (n = 109) and 57% (n = 144) of child participants, respectively. All the participants originated from the western-central Greater Poland Voivodeship. The data analyzed in this study were collected in 2019. Internal consistency of the subscales and correlation between them were measured using Cronbach’s Alpha reliability coefficient and Spearman's correlation coefficient, respectively. Confirmatory factor analysis was conducted for both groups (children and parents) in two-, three- and four-factor models. The confirmatory factor analysis for both groups (children and parents) attributed the four-factor model with the highest goodness-of-fit, fulfilling the criteria of a good-fitting model. The results show that the Multisource Assessment of Children’s Social Competence Scale is an appropriately adapted tool for the evaluation of the social competence of children in Poland, taking different rater perspectives into account.

## Introduction

Social competence is the ability to successfully achieve and maintain positive social outcomes using personal and environmental resources^1–5^. Moreover, Rubin and Rose-Krasnor emphasized the role of social competence in maintaining positive relationships with the environment^6^. They defined social competence as "the ability to achieve personal goals in social interaction while maintaining positive relationships with others over time and in varying situations”^6^ (p. 285). Social competence is a term describing the general adaptation to functioning in the society, while the notion of social skills is usually used to characterize the subjects ability to respond to a particular setting. The former consists of a number of elements, including the ability to take an outside perspective into account in the decision making process, learn from the past experiences, as well as use the obtained knowledge to adapt to the circumstances of the constantly changing social landscape^7^. Hence, a person’s social competence broadly reflects other people’s perception of their ability to succeed in a specific setting^8^.

Social competence is associated with two types of behavior: prosocial and antisocial^4,7^. In this concept, being socially competent means that a child scores high on the aspects of prosocial behavior and low on antisocial behavior^4,7^.

Socially competent children exhibit the ability to work together, solve difficulties, and live in harmony with others^9–11^. Most results of studies on children’s social abilities demonstrate links between low social competence and behavioral deficiencies, such as externalization, conduct problems, and aggression^4,7,12^. Therefore, children and adolescents require more than just education and professional skills to live a successful life, obtain knowledge, and develop their eventual career^4,7,12,13^.

MASCS, partly derived from Merrel and Gimpel’s 1998 School Social Behavior Scale (SSBS), was designed by Junttila, Voeten, Kaukiainen, and Vauras to allow teachers to assess the social behavior of elementary school students effectively. The name of the scale, Multisource Assessment of Social Competence, derives from the instrument’s reliance on four different perspectives (child’s own, peers’, teachers’, and parents’)^4^. MASCS is a multi-component measurement tool of children’s social skills, in the context of school education, accounting for various aspects of both pro- and antisocial behavior.

MASCS it is an international tool with high relevance and reliability^4^. Its adaptation will allow for a comparison of Polish surveys with those conducted in other countries.

There is a gap in the Polish educational context in terms of tools used to examine social competence (including pro-social and anti-social). Hence, there is a need for a tool that is easy to use by children and adolescents, and examines such important aspects as cooperation skills, empathy, impulsivity, disruptiveness.

This study aimed to adapt the Multisource Assessment of Social Competence Scale (MASCS), an instrument to evaluate the social competence of elementary school children, from the perspective of students and their parents, for use in the Polish population. The research was conducted at school, on both children and parents. The research including children took place during computer science lessons, while parents were examined during regular meetings with teachers. Teachers were not included in the research, as it is difficult, in the Polish educational system, to assign individual teachers to a particular year in certain teaching subjects.

The Polish adaptation of the MASCS tool allows it to be used by educators and school psychologists to diagnose students' social and antisocial behavior, especially for students who display undesirable behavior at school, such as conflicts with peers and teachers, aggressive behavior, and exclusion.

As social skills can be reliably measured within specified cultural and language limits, their assessment provides valuable insight, helpful in enhancing learning environments and ensuring their skill-development-friendly status^14^. Effective social skill assessment can provide teachers and parents with information about social and emotional skill deficits and trends, indicate the potential need to modify teaching and parenting strategies, and evaluate the significance of these skills in fostering children’s lifetime success^15–17^.

## Methods

### Participants

The data analyzed in this study were collected, in 2019, from children (group 1, n = 253) and their parents (group 2, n = 248). The study was based on convenient sample. The sample was recruited from public schools. All surveyed elementary school children (fourth through eighth grade) and their parents were of Polish descent, European (Caucasian) ancestry. The difference in the size of the study groups resulted from the lack of evaluation of 5 children by their parents. The parent group was treated as a whole, with 10% of fathers and 90% of mothers included in the study. One parent of each child (mother or father) participated in the study. The data were linked between parents and children. The study group contained 43% (n = 109) boys and 57% (n = 144) girls, aged 9–15, with a mean of 11.85 years (SD = 1.63). In addition, 30 children were subsequently examined to evaluate adapted MASCS compatibility. The participants were recruited from public primary schools in the western-centrally located Greater Poland Voivodeship. The following inclusion criteria were applied for the participating students: age 9–15, attending schools participating in the project, parents’ consent to their children's participation in the surveys. Students from schools in which no one has been included in a structured program to support social skills in the last 6 months were excluded from the study. The only inclusion criterion for parents of children (aged 9–15, from schools participating in the project) was their consent to participate in the survey.

Verbal consent was obtained from the students before taking the survey. The students were informed of the voluntariness of joining the study and the possibility of terminating their consent for participation in the study at any time. Informed written consent for their and their children’s inclusion in the study was obtained from participating parents. Parents of the children were informed of research confidentiality, and the fact that the results of the study would only be used in the form of scientific summary studies, without revealing any personal information. Parent data was collected in a paper (printed) form through an anonymous survey, with a full effort to ensure confidentiality during data collection. In turn, children’s data was collected digitally. During IT classes, the students completed an online questionnaire, available via a hyperlink. The participants were informed that their inclusion in the study was voluntary. The study was conducted according to the Declaration of Helsinki, with its protocol approved by the Bioethics Committee of the Poznan University of Medical Science (approval no 730/19).

### Social competence measurement

Multisource Assessment of Social Competence Scale comprises two prosocial (CO-Cooperating Skills, EM-Empathy) and two antisocial subscales (IM-Impulsivity; DI-Disruptiveness). The questionnaire rating was based on a 4- point Likert-type scale: 1 = never, 2 = rarely, 3 = frequently, 4 = very frequently.

Evaluation of children’s social competence was based on two perspectives, their own and their parents’, as a choice of a singular perspective would need to be justified by the relevant social context and study purpose^18^. The tool was translated from English into Polish and then back-translated by two independent experts with experience in the social science and humanities. Then, a team of researchers consisting of educators, a psychologist, a sociologist, and a health educator with experience in psychological and pedagogical research and practice, compared the translated tool with the original, taking into account the “correctness” of the language and intent of items (Table 1).

**Table 1:** Items in the English and Polish versions of the MASCS.

Item	English version	Polish version
Cooperating skills		
CO1	Offers help to other students	Oferuje pomoc innym uczniom
CO2	Effectively participates in group activities	Efektywnie uczestniczy w zajęciach grupowych
CO3	Invites other students to participate in activities	Zachęca innych uczniów do udziału w zajęciach
CO4	Is skillful in starting conversations with mates	Umiejętnie rozpoczyna rozmowy z kolegami i koleżankami
CO5	Cooperates with other students	Współpracuje z innymi uczniami
Empathy		
EM1	Knows how to be a good friend	Wie, jak być dobrym przyjacielem
EM2	Is sensitive to the feelings of others	Jest wrażliwy/a na uczucia innych
EM3	Shows acceptance of others students	Akceptuje innych uczniów
Impulsivity		
IM1	Has a short fuse	Szybko wpada w złość
IM2	Has temper outbursts or tantrums	Ma wybuchowy temperament lub napady złości
IM3	Is easily irritated	Łatwo się irytuje
Disruptiveness		
DI1	Teases and makes fun of other students	Dokucza innym uczniom i śmieje się z nich
DI2	Argues and quarrels with peers	Kłóci się i sprzecza z rówieśnikami
DI3	Bothers and annoys other students	Denerwuje i wkurza innych uczniów
DI4	Acts without thinking	Działa bezmyślnie

The resulting scale was compared with the original to evaluate the correctness of the translation, with the results proving high accuracy of the adaptation. The adaptation contained 15 items assessed on a four-point scale, rated by both the children and their parents, as in the original. The only difference between the children’s and parents’ forms was the subject of the items (e.g., “I have a short fuse” vs. “Has a short fuse”).

### ICC

The MASCS assessment was conducted on two different dates in 30 participants to evaluate thereliability of the obtained adaptation. Based on the workflow suggested in the 2016 work by Koo and Li, the reliability of the repeated measurements was tested using the interclass correlation coefficient ICC (2,k)^19–21^. Each of the four subscales was attributed with good to excellent reliability, with ICC values of 0.75–0.93. In turn, ICC confidence intervals for CO and EM subscales indicated moderate to good reliability (0.53–0.87), with good to excellent reliability for IM and DI subscales (0.86–0.96).

### Statistical analysis

The results for each scale item, divided by the study groups, were presented using descriptive statistics, such as the mean, standard deviation (SD), skewness and kurtosis. The cutoff values for skewness and kurtosis were assumed at − 2 to 2 and − 7 to 7, respectively^22^.

In turn, Cronbach’s alpha was used to evaluate the reliability (internal consistency) of the four subscales in both studied groups. Due to the characteristics of the Likert scale on which MASCS is based, nonparametric tests were used. Correlations between the subscales, as well as between the scores of individual questionnaire items and the subscale to which they belong, were examined using Spearman’s rank correlation.

Confirmatory Factor Analysis (CFA) was used to formally verify and confirm the factor structure of the studied MASCS concept^23,24^. Confirmation analysis was performed separately for both groups in three models, depending on the number of factors^4,5,25,26^. The two-factor model included the Prosocial and Antisocial scales, the three-factor model was based on one scale (Prosocial), and two subscales (Impulsivity, Disruptiveness), and the four-factor model was divided into all four subscales (Cooperation skills, Empathy, Impulsivity, Disruptiveness).

The various methods for model fit evaluation: model chi-square with its degrees of freedom and P value, Bentler’s Comparative Fit Index (CFI)^27^, Steiger–Lind’s Root Mean Square Error of Approximation (RMSEA)^28^ with its 90% confidence interval, and the Standardized Root Mean Square Residual (SRMR), were previously described by Kline et al.^24^. In this publication, CFI, RMSEA and SRMR were selected as GFC parameters (Goodness of Fit Criterion parameters), with their values indicative of the model’s goodness of fit. Additionally, the Tucker–Lewis Index (TLI)^29^ was used to compare the results with those presented in the original publication describing MASCS^4^.

### CFI

Bentler’s CFI^27^ is an incremental fit index, also serving as a goodness-of-fit statistic. Its values range from 0 to 1, where 1 is the best result. The CFI compares the number of departures from the close fit assumed in the experimental model with the values characteristic of the independence (null) model. While CFI > 0.90 was initially considered representative of a well-fitting model^30^, a cutoff value close to 0.95 has later been advised^31^.

While CFI > 0.90 was considered acceptable in this study (a necessary condition to consider the model well-fitting), the goodness of fit was considered optimal if the CFI values were closer to 0.95.

### SRMR

The SRMR is the average of the standardized residuals between the observed and the predicted covariance matrix. A cutoff value close to 0.08 indicates a good fit^31^ and shouldn’t exceed 0.10, as SRMR > 0.10 indicates a poor fit^23,24^. In this study, 0.08 was assumed as the cutoff value, with SRMR < 0.08 considered indicative of a well-fitting model.

### RMSEA

The RMSEA is an absolute fit index scaled as a badness-of-fit statistic, where zero value indicates the best result. This index was first presented in a 1980 study by Steiger and Lind. The RMSEA takes the error of approximation in the population into account, attempting to answer a question: “How well would the model, with unknown but optimally chosen parameter values, fit the population covariance matrix if it was available?”^32^. This discrepancy, measured by the RMSEA, is expressed per degree of freedom, thus making it sensitive to the number of estimated parameters in the model (i.e., the complexity of the model). RMSEA < 0.05 indicates a good fit, and RMSEA < 0.08 represents reasonable population approximation errors^32^. RMSEA values ranging from 0.08 to 0.10 indicate mediocre fit, and RMSEA > 0.10 are characteristic of a poor fit^33^. Furthermore, a value of 0.06 was suggested to indicate a good fit between the hypothesized model and the observed data^31^. In this study, a RMSEA value of < 0.08 was assumed to be acceptable, a necessary condition to consider the model as well-fitting. Nonetheless, the goodness of fit was considered optimal if the RMSEA value was closer to 0.06.

### TLI

The Tucker–Lewis Index (TLI)^29^, also known as the Non-Normed fit index (NNFI), assumes values between 0 and 1 and indicates the model’s goodness of fit. The cutoff values for TLI are the same as in CFI: TLI > 0.90, optimally close to 0.95.

The significance cutoff value in this study was assumed at *P* < 0.05. All the analyses were conducted using the TIBCO Software Inc. (2017) Statistica (data analysis software system; version 13; available at http://statistica.io) and the jamovi project (2021). *jamovi*. (Version 2.2; [Computer Software]; available at https://www.jamovi.org).

## Results

Table 2 contains the mean and standard deviation (SD) values, skewness and kurtosis for all 15 items, separately for both study groups. The skewness and kurtosis results are within the accepted norms of − 2 to 2 and − 7 to 7, respectively.

**Table 2:** Descriptive statistics results for each MASCS item in both study groups.

Item	Group 1 (n = 253)				Group 2 (n = 248)			
	Mean	SD	Skewness	Kurtosis	Mean	SD	Skewness	Kurtosis
Cooperation skills								
CO1	2.87	0.77	− 0.09	− 0.67	3.23	0.69	− 0.34	− 0.89
CO2	3.01	0.82	− 0.41	− 0.55	3.29	0.71	− 0.63	− 0.31
CO3	2.30	0.94	0.29	− 0.77	2.77	0.81	− 0.14	− 0.54
CO4	3.06	0.87	− 0.62	− 0.36	3.20	0.71	− 0.51	− 0.13
CO5	3.11	0.81	− 0.48	− 0.61	3.36	0.61	− 0.52	− 0.05
Empathy								
EM1	3.43	0.76	− 1.28	1.18	3.52	0.60	− 1.04	1.28
EM2	2.78	1.01	− 0.24	− 1.09	3.53	0.67	− 1.28	1.06
EM3	3.30	0.75	− 0.85	0.17	3.57	0.60	− 1.29	1.76
Impulsivity								
IM1	2.39	0.88	0.47	− 0.51	2.21	0.87	0.58	− 0.19
IM2	1.96	0.92	0.77	− 0.18	1.98	0.88	0.69	− 0.15
IM3	2.39	0.94	0.22	− 0.82	2.15	0.82	0.49	− 0.10
Disruptiveness								
DI1	1.45	0.70	1.75	3.11	1.32	0.55	1.69	2.70
DI2	1.90	0.77	0.70	0.40	1.65	0.62	0.72	1.01
DI3	1.63	0.78	1.15	0.88	1.53	0.62	0.84	0.21
DI4	1.81	0.90	1.02	0.32	1.67	0.75	0.99	0.67

*SD* standard deviation.

Internal consistency of the CO, IM and DI subscales, measured using Cronbach’s Alpha reliability coefficient, ranged from 0.80 to 0.84 in group 1 and from 0.81 to 0.89 in group 2. For the EM subscale, a slightly lower consistency was obtained, α = 0.56 in group 1 and α = 0.75 in group 2 (Table 3).

**Table 3:** Spearman’s correlation coefficients between the subscales, and Cronbach’s alphas, presented separately for both study groups.

Item	CO	EM	IM	DI	Cronbach’s alpha in group 2
CO	–	**r = 0.53 *P* < 0.001**	**r = − 0.27** ***P* < 0.001**	**r = − 0.26** ***P* < 0.001**	**0.81**
EM	*r* = *0.60* *P* < *0.001*	–	**r = − 0.29** ***P* < 0.001**	**r = − 0.40** ***P* < 0.001**	**0.75**
IM	*r* = *− 0.14* *P* = *0.027*	*r* = *− 0.04* *P* = *0.513*	–	**r = 0.52** ***P* < 0.001**	**0.89**
DI	*r* = *− 0.17* *P* = *0.008*	*r* = *− 0.20* *P* = *0.002*	*r* = *0.45* *P* < *0.001*	–	**0.84**
Cronbach’s alpha in group 1	*0.80*	*0.56*	*0.84*	*0.80*	–

*Group 1 – results*, **Group 2 – results.**

*CO* Cooperating Skills, *EM* Empathy, *IM* Impulsivity, *DI* Disruptiveness.

In both groups, significant (*P* < 0.05) positive correlations were detected between the subscales inside the particular scales (CO vs EM and IM vs DI). The correlations coefficients were 0.60 (CO vs EM) and 0.45 (IM vs DI) in group 1 and 0.53 and 0.52 in group 2, respectively. In turn, correlation coefficients between subscales from different scales (CO vs IM, CO vs DI, EM vs IM and EM vs DI) were negative in both study groups. In the first group, the correlations between CO and IM, CO and DI, and EM and DI were significant (*P* < 0.05) and ranged from − 0.20 to − 0.14. In the second group, all correlations between subscales of different scales were significant (*P* < 0.05), ranging from − 0.40 to − 0.26 (Table 3).

Confirmatory factor analysis (CFA) was conducted for both groups in three models, depending on the number of factors. The two-factor model, for both groups, did not meet the goodness of fit criteria based on both CFI (< 0.90) and RMSEA (> 0.08) (Table 4).

**Table 4:** Results of the confirmatory factor analysis of MASCS, presented separately for both study groups.

	χ^2^ (df)	CFI	TLI	RMSEA (90% CI)	SRMR
Two-factor (Prosoc, Antisoc)					
Group 1 (n = 253)	333.52* (89)	0.817	0.784	0.104 (0.092–0.116)	0.079
Group 2 (n = 248)	509.08* (89)	0.757	0.713	0.138 (0.126–0.150)	0.105
Three-factor (Prosoc, IM, DI)					
Group 1 (n = 253)	181.15* (87)	0.930	0.915	0.065 (0.052–0.079)	0.063
Group 2 (n = 248)	265.36* (87)	0.897	0.875	0.091 (0.079–0.104)	0.075
Four-factor (CO, EM, IM, DI)					
Group 1 (n = 253)	173.02* (84)	0.933	0.917	0.065 (0.051–0.078)	0.062
Group 2 (n = 248)	193.51* (84)	0.937	0.921	0.073 (0.059–0.086)	0.063
Goodness of fit criterion		> 0.90	> 0.90	< 0.08	< 0.08

*CFI* Comparative Fit Index, *TLI* Tucker-Lewis Index, *RMSEA* Root Mean Square Error of Approximation, *SRMR* Standardized Root Mean Square Residual.

*P < 0.001. χ^2^ (df), Model chi-square (degrees of freedom).

In the case of the three-factor model in group 1, the GFC parameters indicate a good fit of the model (CFI = 0.930, RMSEA = 0.065, SRMR = 0.063). In turn, the three-factor model for group 2 did not meet the goodness of fit criteria due to too low CFI (< 0.90) and too high RMSEA (> 0.08) (Table 4).

In both groups, the four-factor model meets the goodness-of-fit criteria for all GFC-parameters for group 1 (CFI = 0.933, RMSEA = 0.065, SRMR = 0.062) and group 2 (CFI = 0.937, RMSEA = 0.073, SRMR = 0.063).

## Discussion

The study aimed to develop a Polish adaptation of the Multisource Assessment of Children’s Social Competence Scale (MASCS), used to measure social competence from both the children’s and their parents’ perspectives. The goodness of fit for the adapted scale was examined using confirmatory factor analysis models.

The two-factor model evaluated the Prosocial and Antisocial scales. The results proved that it was not well-fitting for both children’s and parents’ groups, as evidenced by the values of GFC parameters. Similar outcomes were previously reported in the study of Vuori et al.^26^ (CFI = 0.86 for children’s, CFI = 0.70 for mothers’ and CFI = 0.77 for fathers’ groups). Contrarily, the authors of the scale found this model to be well-fitting in the children’s study group (NNFI = 0.95), but not in the parents’ group due to too high result of SRMR (SRMR = 0.088)^4^.

The three-factor model was based on one scale (Prosocial) and two subscales (Impulsivity, Disruptiveness). This model proved to be a good fit for the children’s group in our study, confirming the findings of Junttila et al.^4^ (NNFI = 0.91) and Vuori et al.^26^ (CFI = 0.93). In turn, the model did not exhibit similar goodness of fit in the parents’ group as described in the research of the authors mentioned above, (NNFI = 0.84 for parents^4^, CFI = 0.83 for fathers and CFI = 0.85 for mothers^26^).

The four-factor model comprised all the subscales: Cooperation skills, Empathy, Impulsivity, and Disruptiveness. This model was better fitting than its two- and three-factor counterparts, both in our studies and previous reports of Junttila et al.^4,5,25^ and Vuori et al.^26^. The goodness of fit is evidenced in the best GFC-parameter values in both studied groups. In all the publications mentioned above, except for the 2017 study by Vuori et al.^26^, in which the model exceeded the cutoff RMSEA value for mothers (RMSEA = 0.082) and fathers (RMSEA = 0.091), all GFC parameters met the goodness of fit criteria.

In previous studies using the MASCS, all participating children were students of the same school year, such as fourth-grade students^4,5,25^, fourth-grade students, with a second sample of 13-years-old children^5^, or sixth-grade students^34^. In contrast, our study was based on students representing a broader age range, attending fourth to eighth grade (ages 9–15). The age range matched the original study, with the assumption to study children in an comparableage range , rather than in individual years. The results confirm that the Multisource Assessment of Children’s Social Competence Scale is an appropriately adapted tool for the evaluation of the social competence of elementary school students.

The limitations of our research include a smaller number of participants in both group 1 (n = 253) and group 2 (n = 248) compared to the original study^4^, in which the sample size was n = 446 in the first n = 445 in the second group, or subsequent studies of the same scale, with sample sizes of n = 318^26^, or n = 981^25^. Despite the smaller number of participants, our four-factor model fulfilled the assumed goodness of fit criteria. The sample size is smaller than in the original research, but still meets the conditions necessary (n > 100) to conduct a confirmatory analysis of the studied tool.

Another limitation is the lack of peer and teacher evaluations found in other research. Therefore, the plan is to include these assessments and expand our sample size in subsequent studies.

## Conclusions

The present study confirms that the Multisource Assessment of Children's Social Competence Scale is an appropriately adapted tool for assessing the social competence of elementary school students. The tool can be applied as a way to diagnose the level of students' competence and their possible needs in this aspect. The MASCS tool makes it possible to assess the level of students' social and antisocial behavior and foster preventive measures. Diagnosis of antisocial behavior can be helpful in designing tailored psychological and pedagogical intervention for students. Therefore, it is important to identify the right area for working with students with relationship difficulties, which can help to mitigate undesirable behavior, and ultimately integrate excluded students with their peers, reduce conflict, and eliminate aggressive behavior. Appropriate diagnosis and appropriately targeted measures for the development of social competence will contribute to the well-being and mental health of students^35,36^. Measuring the level of social competencies and deficits will also allow the inclusion of preventive pedagogical measures aimed at the holistic development of students (not only educational, but also mental and social).

In future studies, it can also serve as a tool to measure the effectiveness of the intervention programs conducted to develop students' social competencies^37^.With the adaptation of the MASCS into the Polish language, it will be possible to conduct further scientific studies assessing the level of social competencies and compare them to the results of other countries. Overall, based on the results of this study, we consider thie tool to be successfully adapted.

## Data Availability

The data generated and/or analyzed during the current study are available from the corresponding author on reasonable request.
